# Effect of 1-MCP and KMnO4 treatments with different packaging on quality preservation of golden delicious apples

**DOI:** 10.1016/j.fochx.2024.101768

**Published:** 2024-08-24

**Authors:** Aaruba Maqbool, Mushtaq Ahmad Beigh, Syed Zameer Hussain, Tashooq Ahmad Bhat, Imtiyaz Ahmad Zargar, Shazia Akhter, Nazrana Wani, Tahiya Qadri

**Affiliations:** Division of Food Science and Technology, Sher-e-Kashmir University of Agricultural Sciences and Technology (SKUAST) Kashmir, Shalimar, 190025, India

**Keywords:** Apple cv. golden delicious, 1-Methylcyclopropene, Potassium permanganate, Packaging: Fruit quality preservation: rot incidence reduction

## Abstract

This study explored the impact of three packaging materials (wooden boxes, corrugated fiber boxes, shrink-wrapped boxes) combined with two ethylene scrubbers (1-MCP, KMnO_4_) on the shelf life of Golden Delicious apples. While previous research has extensively studied the effects of packaging and ethylene inhibitors independently, the novelty of this work lies in its combined evaluation of these factors under ambient storage conditions over an extended period of 160 days. The study specifically addresses a research gap by directly comparing the efficacy of 1-MCP and KMnO_4_ within different packaging environments, offering insights into their combined influence on key quality parameters such as firmness, juice yield, rot incidence, physiological loss in weight (PLW), acidity, and total soluble solids (TSS). Findings revealed that 1-MCP-treated apples, particularly when shrink-wrapped, experienced minimal reductions in firmness and juice yield, with significantly lower rot incidence and physiological loss in weight (PLW) compared to KMnO_4_-treated and control apples. Additionally, while acidity and juice content naturally declined over time, and TSS initially increased before decreasing, 1-MCP-treated apples exhibited more stable quality attributes. The study also noted a slower decline in organoleptic quality with 1-MCP and shrink-wrap packaging. The research concludes that the combination of 1-MCP treatment and shrink-wrap packaging most effectively extends the shelf life of Golden Delicious apples, highlighting the importance of integrated approaches to post-harvest management. This study provides a novel framework for improving storage techniques, particularly for ambient conditions where shelf life extension is most challenging.

## Highlights

• 1-MCP treated apples showed minimal firmness and juice yield reduction over 160 days.
• Control and KMnO4 treated apples exhibited higher rot incidence and weight loss.
• Acidity and juice content decreased during storage across all treatments.
• 1-MCP treated apples maintained better organoleptic qualities, especially in shrink-wrapped boxes.
• The combination of 1-MCP treatment and shrink-wrap packaging effectively extended the shelf life of Golden Delicious apples.

## 1 Introduction

Apples, originating from Asia, are now cultivated worldwide across diverse climates, from temperate to tropical regions. They are commercially grown in over 63 countries within latitudes of 25° to 52°, benefiting from various growing conditions and involving numerous cultivars (Lauri & Simon, 2019: Musacchi & Serra, 2018). Apples rank as the fourth most significant fruit crop globally, following citrus fruits, grapes, and bananas (Assouguem et al., 2024). The annual average global apple production for 2023/24 is forecast to edge slightly higher up 175,000 MT to 84.6 million tons (USDA, 2023). China stands as the leading producer, contributing a staggering 48 % of the world's total apple production. The United States is the second-largest producer, accounting for 6.1 % of the total, followed by Poland (3.8 %), India (2.9 %), Turkey (2.9 %), and Italy (2.9 %) making significant contributions. This distribution underscores the widespread cultivation, consumption, and economic importance of apples on a global scale. In India, Jammu and Kashmir (J&K) holds a prominent position in area and production of apples with the production of 1901.85 metric tonnes in 2021–2022 (National Horticulture Board).

Apples are considered highly nutritional and are valued in a healthy diet due to their composition. They contain over 80 % water, sugars (with fructose dominating over glucose and sucrose), organic acids (ranging from 0.2 % to 0.8 %), vitamins (primarily vitamin C, with levels between 2.3 and 31.1 mg/100 g dry matter), minerals (ash content ranging from 0.34 % to 1.23 %), and dietary fibers (approximately 2–3 %, with pectin constituting less than 50 % of apple fibers) (Szpadzik, Molska-Kawulok, Krupa, & Przybyłko, 2023). Additionally, apples account for 10–30 % of daily fiber and potassium intake (Kumar, 2023). The main phenolic compounds found in apples include chlorogenic acid, epicatechin, procyanidins, phloretin, and quercetins. The specific profile of these compounds depends on factors such as variety of apple, environmental conditions, agricultural techniques, maturity stage, and storage period (Korban, 2023).

Over time, the quality of fruits and vegetables naturally declines after being harvested. Due to their highly perishable nature, these commodities are susceptible to post-harvest losses, presenting a notable obstacle to ensuring consumer access to these nutritious products. Post-harvest operations, particularly packaging, play a crucial role in addressing this issue. As a vital component of the supply chain, packaging significantly contributes to extending the shelf life of perishable items like fruits and vegetables. It ensures to secure vegetables and fruits during handling, shipping, and storage after harvest assuring that the products remain available to consumers in optimal condition. The primary purpose of packaging is to shield the packaged food items from various external factors that can lead to deterioration, including light, heat, pressure, dust, dirt & microorganisms.

One effective post-harvest approach for handling fruits and vegetables is shrink-wrap packing, an innovative technique involving the tight wrapping of produce in heat-shrinking plastic film to create a protective seal. This technique helps reduce moisture loss, prevent physical damage, and extend shelf life by preserving freshness. The clear packaging also allows consumers to visually assess product quality. However, the environmental impact of plastic packaging should be considered, and sustainable alternatives explored.

Another is cardboard packaging, specifically in the form of corrugated fiber boxes (CFB), stands out as a fundamental material extensively employed in the horticultural industry. Corrugated fiber boxes (CFB), is widely used in the horticultural industry due to its light weight, durability, and ease of handling. CFB (corrugated fiber boxes) consist of a corrugated paper medium sandwiched between two liners, providing strong stacking strength. They effectively protect fruits and vegetables from mechanical forces and vibrations during transit, with the option to separate items using bubble wrap or trays in multi-layer designs. Studies confirm CFB's (corrugated fiber boxes) effectiveness in preventing physical damage to apples, including the use of 7-ply with a 10 kg capacity for long-distance transportation.

Wooden boxes and crates, known for their strong stacking strength and durability, are often used for packing fruits, especially apples. However, the higher cost of wood has raised concerns, leading to a shift toward alternative materials. Research by Njume, Ngosong, Krah, and Mardjan (2020) highlighted that wooden boxes can cause physical damage to fruits during storage and transit. A linear relationship was found between mechanical stress and damage in Granny Smith, Golden Delicious, and Stark Spur apples, with significant differences in damage between the top and bottom layers of fruit.

Two key postharvest methods to extend apple shelf life include using the ethylene inhibitor 1-methylcyclopropene (1-MCP) and the ethylene absorber potassium permanganate (KMnO₄). Cold storage slows respiration and reduces ethylene production, while 1-MCP prevents ethylene-induced ripening by binding to ethylene receptors. This binding blocks the signaling pathway that normally leads to fruit ripening, allowing apples to stay fresh longer. KMnO₄ absorbs ethylene in the storage environment, further delaying ripening and extending the fruit's shelf life (Lee & Yoon, 2017: Dias et al., 2021).

Potassium permanganate (KMnO₄), a purple crystalline substance with strong oxidizing properties, is highly effective in reducing ethylene levels in fruit storage environments. When KMnO₄ oxidizes ethylene (C₂H₄), it sequentially transforms the molecule: ethylene is first converted to acetaldehyde (CH₃CHO), then to acetic acid (CH₃COOH), and finally, under more intense conditions, to carbon dioxide (CO₂) and water (H₂O). This oxidation process significantly lowers ethylene concentrations, slowing fruit ripening and extending shelf life, thereby preserving fruit quality for longer periods (Kumar et al., 2024).

This research fills a gap in understanding how integrated packaging and ethylene control strategies can be optimized for fruit storage, providing valuable insights for both the industry and consumers. Thus, the aim of the study is to assess the impact of wooden boxes, corrugated fiber boxes, and shrink-wrapped boxes on the quality and shelf life of Golden Delicious apples, to examine the effects of ethylene scrubber's 1-methylcyclopropene (1-MCP) and potassium permanganate (KMnO₄) on apple quality in conjunction with the different packaging materials. Measure changes in firmness, juice yield, rot incidence, physiological loss in weight (PLW), acidity, juice content, and total soluble solids (TSS) during a 160-day storage period. Analyse the sensory attributes of the apples over time to determine the impact on overall organoleptic quality and to determine the most effective combination of packaging material and ethylene scrubber for prolonging the shelf life and maintaining the quality of Golden Delicious apples.

## 2 Material and method

### 2.1 Procurement of raw material

Golden Delicious apples were procured from the high-density orchards of Sher-e-Kashmir University of Agricultural Sciences and Technology, Kashmir (SKUAST-K). To ensure a representative sample, apples were randomly selected from various trees across the orchard. A harvest of 200 kg of apples was collected and pre-cooled under shade to maintain their freshness, before being packed in different packaging materials, including wooden boxes, corrugated fiberboard (CFB) boxes, and shrink-wrapped boxes. The apples were then subjected to three distinct treatments: T1 (Control), where no treatment was applied; T2, where the apples were treated with 1 ppm 1-MCP (SmartFresh) cards; and T3, where the apples were treated with 5 g KMnO_4_ sachets. Afterwards, the treated and packaged apples were stored under ambient conditions (18 ± 5 °C, RH 60 %) for 160 days to assess the quality attributes.

#### 2.1.1 Preparation of raw material

The apples were stored in three different packaging materials procured from the local market viz., Wooden boxes (20 × 12 × 4), 3 ply Corrugated fiber boards (CFB) (18 × 12 × 12) and Shrink warp (25 μm), and then were subjected to 1-Methylcyclopropene (1-MCP) and potassium permanganate (KMnO_4_) treatments. Control samples were also maintained from each packaging type, without any chemical treatment. The apples were kept under ambient conditions (*T* = 18 ± 5 °C; RH = 60 %) for a storage period of 160 days to evaluate the various physico-chemical attributes at an interval of 20 days. The chemical reagents used in the analysis were procured from Sigma Aldrich, USA.

### 2.2 Physical analysis

#### 2.2.1 Starch iodine rating (1–6 point scale)

Starch iodine rating is a maturity index that determines the conversion of starch to sugars. The 6 point scale was employed to assess the disappearance of starch from the fruit. An iodine solution was prepared by dissolving 4 g of KI (Potassium Iodide) in 400 ml of distilled water, supplemented with 1 g of iodine. Apples were halved along the equator and immersed in the iodine solution for 1 min, followed by a 2-min rest period after removal. Each slice was then swiftly rinsed with clean water, and starch content was estimated using a Generic chart scoring system ranging from 1 (highest starch content) to 6 (lowest starch content) (Wani et al., 2023).

#### 2.2.2 Firmness (lbsq)

Firmness was measured using TA-XT2i Texture analyzer (Stable Microsystems,United Kingdom). The compression probe (50 mm diameter, aluminum cylinder) was applied on single unit of apple to measure compression force. Testing conditions were: 1.5 mm/s pretest speed; 1.5 mm/s test speed; 10 mm/sposttest speed and 5 mm compression distance. (Bhat, Hussain, Naseer, Hameed Rather, & Ahmad Mir, 2021)

#### 2.2.3 Physiological loss in weight (PLW) (%)

For determining Physiological loss in weight (PLW), the weight of fruits was recorded at an interval of 20 days. Physiological loss in weight (PLW) was calculated as per following equation and results were expressed in per cent:

$$\mathrm{PLW}\;\left(\%\right)=\frac{\mathrm{Original weight}-\mathrm{subsequent weight}\;\mathrm{at}\;\mathrm{analysis}}{\mathrm{original weight}}\times 100$$

#### 2.2.4 Rot incidence (%)

Percent rot was examined visually and was calculated using the formula:

$$\mathrm{Rot}\;\text{incidence}\;\left(\%\right)=\frac{\text{Number of diseased fruits}}{\text{Total number of fruits}}\times 100$$

### 2.3 Chemical analysis

#### 2.3.1 Juice Recovery (%)

The juice recovery of apples subjected to different treatments in selected packaging materials were calculated using the formula mentioned:

$$\text{Juice recovery}\;\left(\%\right)=\frac{\text{Weight of juice fruit}}{\text{Average weight of fruit}}\times 100$$

#### 2.3.2 Total soluble solids (^0^Brix)

Total soluble solids (TSS) was measured by a hand-held refractometer (Atago N, Japan) of range 0–32 ^**0**^ Brix and the readings obtained were expressed as ^**0**^ Brix at 20 ^°C^ using reference table (Bhat et al., 2022).

#### 2.3.3 Titratable acidity (% malic acid)

Titratable acidity was determined by following standard AOAC (2005) procedure. Titrable acidity was expressed as percentage malic acid and calculated as per equation:

$$\mathrm{Titratable acidity}\;\left(\%\mathrm{malic acid}\right)=\frac{\begin{array}{c} \text{Titre}\;x\;\text{Normality of alkali}\;x\;\mathrm{Vol}.\text{make}\;\mathrm{up}\;x\;\\ \text{Equivalent weight of acid} \end{array}}{\begin{array}{c} \;\mathrm{Vol}.\text{of sample taken for estimation}\;x\;\text{Weight or}\;\\ \text{volume of sample taken}\;x\;1000 \end{array}}\times 100$$

#### 2.3.4 Texture attributes

Texture analyzer (TX TA.HD. Plus stable Microsystems, England) was used to determine the texture of fruit in terms of bio-yield point, flesh firmness and stickiness. The stainless steel probe P/5 (5 mm diameter) was used during analysis. The instrument was operational at a pre- test and post-test speed of 1.50 mm/s and 10.00 mm/s respectively. The distance between the probe and sample was kept constant at 10 mm. The load cell capacity of the instrument was 250 kg (Bhat, Lone, Shafiq, & Rather, 2021).

### 2.4 Organoleptic evaluation

#### 2.4.1 Sensory evaluation (1–5 point scale)

Sensory evaluation of apples treated with 1-MCP (1-methylpropene) and KMnO_4_ (potassium permanganate) stored in different packaging materials was performed by a panel of 15 semi-trained members from the Division of Food Science and Technology, SKUAST-K, J&K, India. The samples were evaluated for texture, flavor and colour and rated on 5 point scale. Apples were washed and presented to the panel at room temperature under good illumination in sensory booth. The samples for sensory evaluation were coded randomly with three digit numbering to avoid biasness and the results were finally calculated in terms of overall acceptability.

### 2.5 Statistical analysis

The data was statistically analysed through OPSTAT-software using Completely Randomized Design (CRD) in factorial experiment. Experiments were performed in triplicates and data recorded as mean ± SD (standard deviation).

## 3 Results and discussion

### 3.1 Effect of 1-MCP, KMnO_4_ and packaging material on starch iodine rating and firmness

**Fig. 1:**
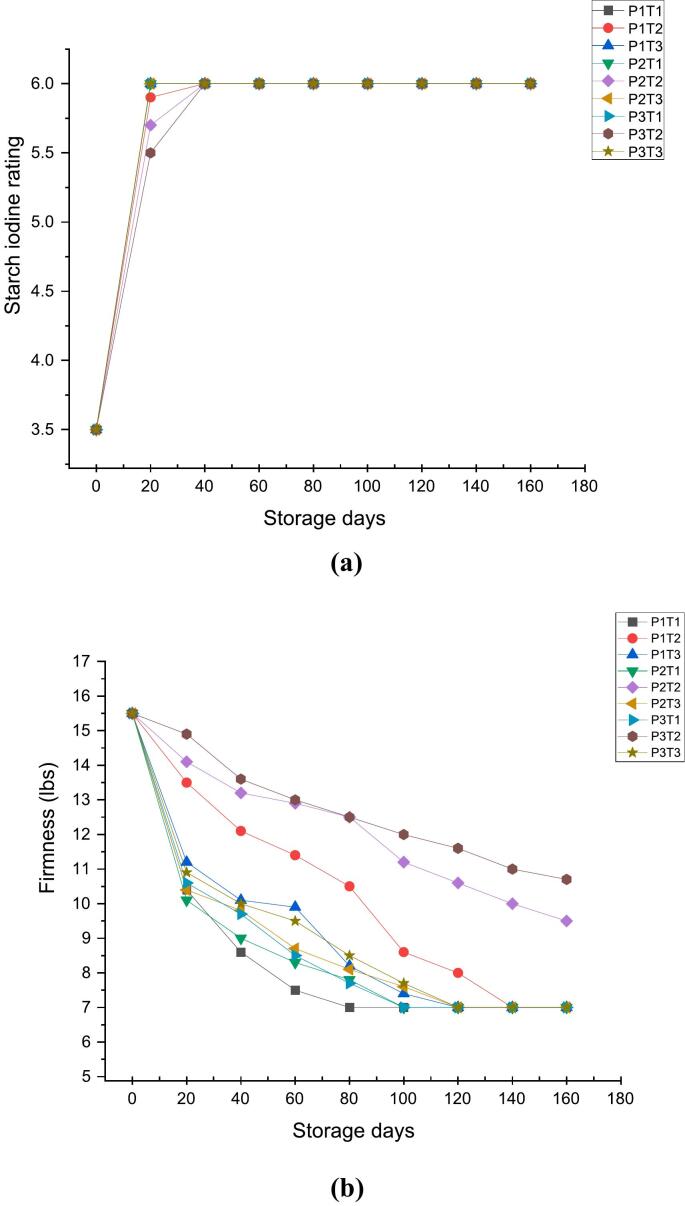
1a Effect of 1-MCP, KMnO_4_ and packaging material on starch iodine rating. 1b: Effect of 1-MCP, KMnO_4_ and packaging material on firmness lb./inch^2^.

The fundamental elements that composes the fruit cells are starch & sugars. The growth and tension of fruit cells as well as the postharvest ripening process are impacted by changes in their contents and components (Yuan et al., 2023).The samples treated with 1-MCP and KMnO_4_ along with the control stored in three different packaging materials were analysed for a storage period of 160 days. The results present in Fig. 1. 1aa depict that there was a significant decrease in starch content of apples which is represented by higher values of starch iodine rating. The starch iodine rating of 3.5 at 0 day increased to 6.00 at 20th day in case of P1TI (wooden box + control), P1T3 (wooden box + KMnO_4_), P2T1 (CFB + control), P2T3 (CFB + KMnO_4_), P3T1 (shrink wrap + control) and P3T3 (shrink wrap + KMnO_4_) respectively. In contrast, the P1T2 (wooden box +1-MCP), P2T2 (CFB +1-MCP) and P3T2 (shrink warp +1-MCP) showed the starch iodine rating of 5.9, 5.7 and 5.5 at 20th day of storage. As illustrated in Fig. 1. 1aa, a notable degradation in starch content was observed across all samples starting from day 11. However, in the case of P3T2, the degradation of starch was notably delayed, and significant levels were retained until the 20th day. This unique trend suggests a potential influence of the specific combination of packaging material (P3) and chemical treatment (T2) in preserving the starch content of the apples during the initial storage period. The reduction in starch content observed during storage can be attributed to its conversion into sugars (Kucuker & Aglar, 2021). These metabolic respiration processes require a readily available source of energy, which is provided by the sugars formed from the breakdown of starch. Consequently, the starch reserves in the fruit diminish as they are systematically converted to sugars to meet the increasing energy demands of these physiological processes, facilitating the ongoing maturation and ripening of the fruit during storage (Brizzolara, Manganaris, Fotopoulos, Watkins, & Tonutti, 2020). Similar results have been reported by Yuan et al. (2023) in Orin apples in which starch degradation is achieved through the combined action of various enzymes, including α-amylase and β-amylase. Wani et al. (2023) also observed a significant reduction in starch content in three different apple cultivars, as the storage duration increased. These results were also in accordance with the findings of Kassebi, Farkas, Székely, Géczy, and Korzenszky (2022), who observed that when ‘Golden Delicious’ apples were kept at room temperature, metabolic mechanisms rapidly converted starch into sugar, leading to a gradual increase in the fruit's sweetness.

Firmness is a critical attribute used to assess the freshness and overall quality of fruits. A firmer fruit generally signifies slower metabolic processes and reduced rates of evaporation, which are indicators of freshness (Ahmad, Zaidi and Arshad, 2021a, Ahmad, Zaidi and Arshad, 2021b). Among the texture qualities that signal the interior quality of other characteristics like crispness, mealiness and hardness is firmness (Fathizadeh, Aboonajmi, & Beygi, 2020). The fruit firmness of 15.5 lb./s at the 0 day of storage decreased to 7.00 lb./sq. for P1T1 (wooden box + control), P1T2 (wooden box +1-MCP) P1T3 (wooden box + KMnO_4_), (CFB + control), P2T3 (CFB + KMnO_4_), P3T1 (shrink wrap + control) and P3T3 (shrink wrap + KMnO_4_) (Fig. 1. 1ab). However, in case of P2T2 (CFB +1-MCP) and P3T2 (shrink warp +1-MCP) the maximum firmness values of 9.5 and 10.7 lb./sq. at 160 days of storage was recorded respectively. The loss in firmness is associated with the progression of ripening. As ripening advances, cells lose water, and their walls become thinner, resulting in reduced firmness. The decrease in water content causes the cells to become less turgid, while the thinning of cell walls weakens their structure. These changes contribute to the overall softening of the fruit during the ripening process (Damarla & Rao, 2023). Additionally, fruit ripening and development alter pectin, hemicellulose and cellulose content which include the conversion of protopectin to water-soluble pectin, breakdown of the middle lamella and cell wall, loosening of hemicellulose and cellulose, causes reduction in intercellular adhesion, enlarges cell gaps, softening of fruit, driven by the high activity of the enzyme endopolygalacturonase and hence decreases firmness (Li et al., 2023: Ahmad, Zaidi and Arshad, 2021a, Ahmad, Zaidi and Arshad, 2021b: Ding et al., 2024: Chen et al., 2023).

The application of 1-MCP along with shrink wrap packaging (P3T2) showing least starch degradation by reducing the expression of β-amylase and high firmness may be due to slow down of enzyme activity which delays the respiratory increase in apples by suppressing the action of internal ethylene concentration Thabiso Kenneth Satekge and Lembe Samukelo Magwaza (2022). Further, shrink wrap prevents the loss of hydrostatic pressure resulting from respiration process and moisture loss thereby, maintains quality attributes (Maqbool et al., 2024).

#### 3.1.1 Effect of 1-MCP, KMnO_4_ and packaging material on physiological weight loss and rot (%)

**Fig. 2:**
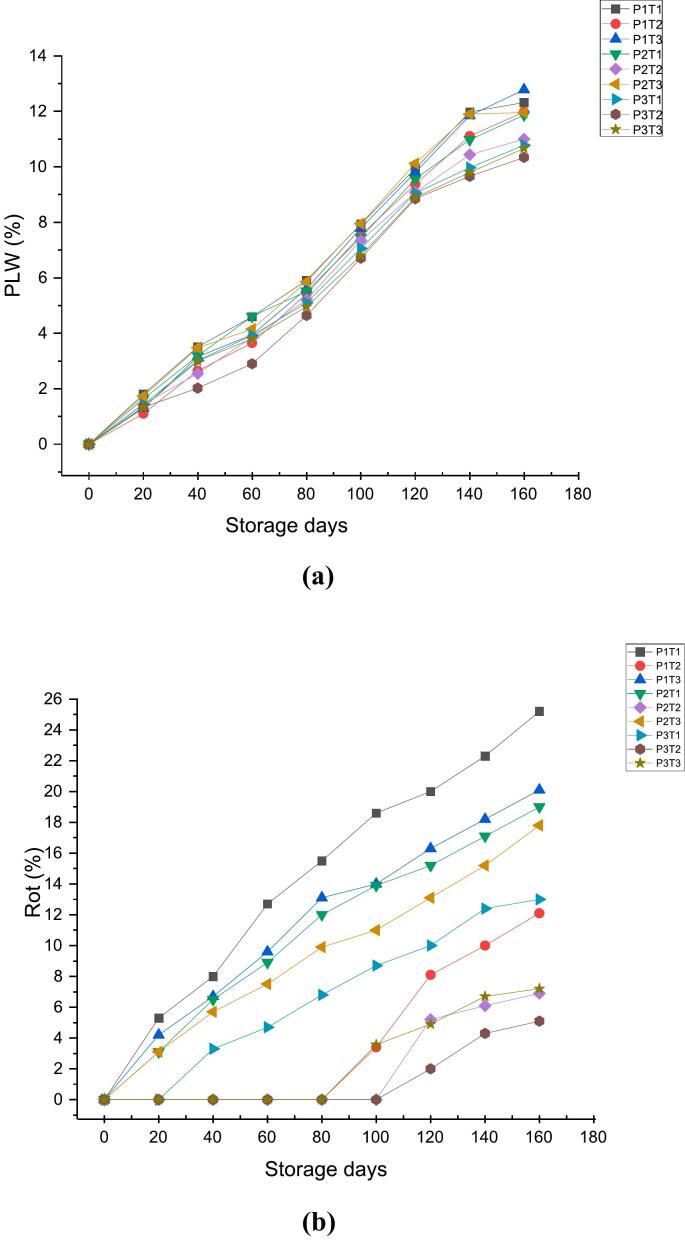
2a: Effect of 1-MCP, KMnO_4_ and packaging material on PLW (%). 2b: Effect of 1-MCP, KMnO4 and packaging material on Rot (%).

Physiological loss in weight (PLW) during storage is a crucial factor influencing their quality and shelf life. The data shown in Fig. 2. 2aa, representing the storage period of 160 days, indicates that apples treated with 1-MCP, KMnO_4_, as well as the control group, exhibited a gradual increase in physiological weight loss (PLW) across the three different packaging materials. This rise is attributed to the continuous transpiration and respiration of moisture from the fruit (Hasan, Singh, Shah, Kaur, & Woodward, 2024). Ethylene accelerates ripening and boosts metabolic activity, which, in turn, elevates both transpiration and respiration rates, leading to increased moisture loss (Hazarika, Lalrinfeli, Lalchhanmawia, & Mandal, 2019).Similar observations have been reported by Chen et al. (2023) who reported continuous increase in physiological loss in weight in Fuji apples during whole storage period. Kassebi et al. (2022) have also observed similar results in Golden Delicious apples with loss percentage of 22.51 and 25.32 % in the fifth and sixth weeks of storage, respectively. Additionally, comparable observations proved by Dutta Roy, Asaduzzaman, Saha, and Khatun (2023) in Guava showed increased Physiological loss in weight (PLW) throughout the storage period with highest weight loss in control sample (14.17 %) compared with coated ones (6.36 %).

The cuticle layer serves as a protective shield, limiting direct contact between pathogens and the underlying fruit tissues (Kumari, Sharma, & Singh, 2019). However, when fruit is bruised typically from excessive or uneven pressure caused by dropping, bumping, or squeezing, the tissue beneath the skin turns brown due to physical damage, compromising this protective barrier (Ahmadi, Barikloo, & Kashfi, 2016). In Fig. 2. 2ab and S1, on visual observations, rot % of 0.0 % at the 0 day of storage increased to 25.2 % for P1T1 (wooden box + control), 20.1 % for P1T3 (wooden box + KMnO_4_), 19.0 % for P2T1 (CFB + control), 17.8 % for P2T3 (CFB + KMnO_4_), 13.0 % for P3T1 (shrink wrap + control) and 7.2 % forP3T3 (shrink wrap + KMnO_4_). However, in case of P1T2 (wooden box +1-MCP), P2T2 (CFB +1-MCP) and P3T2 (shrink warp +1-MCP) the minimum rot % values of 12.1 %, 6.9 % and 5.1 % at 160 days of storage were recorded respectively. Excessive pressure on fruit, from dropping or squeezing, damages the cell walls, causing fluids to ooze out which results in oxidation, leading to browning and degradation. Moreover, the broken cells and exposed fluids create conditions that favour the entry and proliferation of pathogens causing further decay, spoilage, and rot (Stopa, Szyjewicz, Komarnicki, & Kuta, 2018). Furthermore, increased respiration and transpiration accelerate moisture loss, further weakening the fruit and creating favourable conditions for pathogens to proliferate (Jahangir, Makroo, Showkat, & Darakshan Majid, 2022).

The results indicate that 1-MCP proved to be more promising in exerting ethylene inhibition by binding with the ethylene receptors present on the membrane of Endoplasmic reticulum thereby, blocking the downward signaling and transduction (Klein et al., 2019) hence, slowing down the enzyme activity and delays the respiratory increase by suppressing the action of internal ethylene concentration (Ferrante, 2023) whereas, high protection against moisture and oxygen transfer through shrink wrap also prevents fruit degradation as shrink wrap have low WVTR (5.6 cm^3^/m^2^ /24h) and OTR (6313.7cm^3^/m^2^ /24h) (Darwish et al., 2023).

### 3.2 Effect of 1-MCP, KMnO_4_ and packaging material on juice recovery, TSS ^o^Brix, acidity (mallic acid %)

**Fig. 3:**
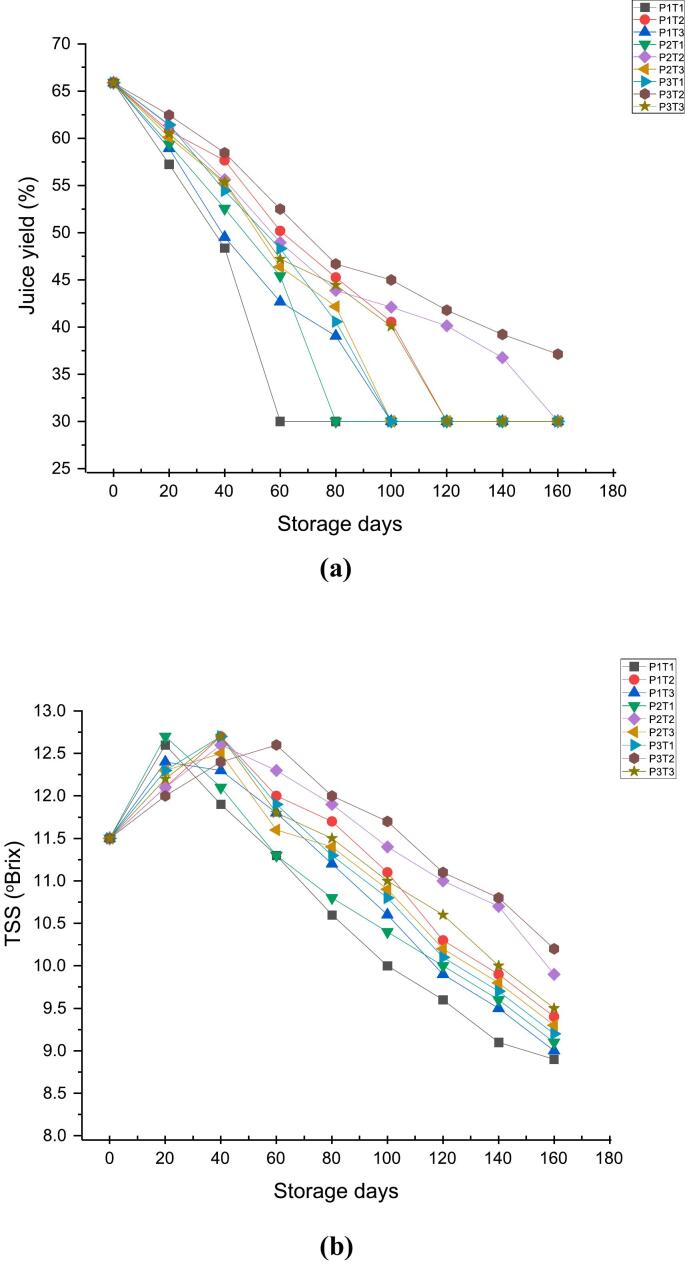

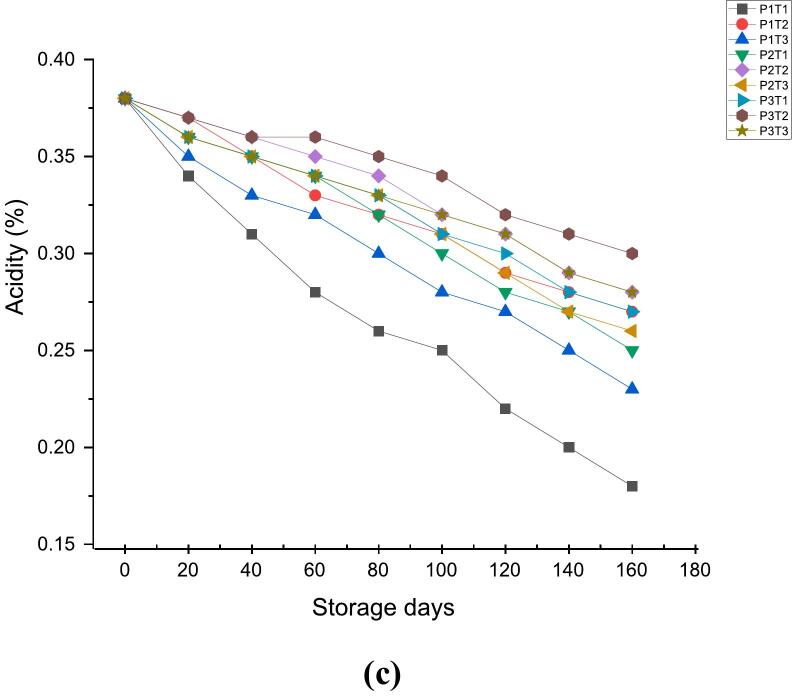
3a: Effect of 1-MCP, KMnO_4_ and packaging material on Juice Yield Recovery (%). 3b: Effect of 1-MCP, KMnO4 and packaging material on TSSOBrix. 3c: Effect of 1-MCP, KMnO4 and packaging material on acidity (mallic acid %).

The effect of 1-MCP, KMnO_4_ and packaging material along with control on chemical parameters (juice recovery, Total soluble solids (TSS) ^o^Brix, acidity as mallic acid %) are shown in Fig. 3. 3aa, 3. 3ab & 3. 3ac. Juice recovery values of 65.88 at 0 day showed sharp decline to 30.00 at 160th day in case of P1TI (wooden box + control), P1T2 (wooden box +1-MCP), P1T3 (wooden box + KMnO_4_), P2T1 (CFB + control), P2T3 (CFB + KMnO_4_), P3T1 (shrink wrap + control) and P3T3 (shrink wrap + KMnO_4_) respectively. In contrast, the P2T2 (CFB +1-MCP) and P3T2 (shrink warp +1-MCP) showed the juice recovery values of 36.75 and 37.12 respectively at 160th day of storage. The decreased rate of juice per cent are in the line with Toy, Lu, Huang, Matsumura, & Liu, 2022 who reported that due to the excessive degradation of pectin in middle lamella by methyl pectin esterase and polygalacturonase enzymes results in separation of the cells from one another leading to the fruit mealy. Jan and Rab (2012) observed a decline in apple juice content, decreasing from 59.27 % to 43.3 % over a 150-day storage period. Wani et al. (2024) also noted a significant decrease in apple juice content under both ambient and cold storage in which after 190 days, juice content dropped to 32.28 % for Gala Redlum, 32.39 % for Red Velox, and 31.35 % for Super Chief in ambient storage, and to 30.49 % for Gala Redlum, 30.49 % for Red Velox, and 30.47 % for Super Chief in cold storage.

Total soluble solids (TSS) content encompasses fruit sweetness and overall flavor profile, playing a crucial role in assessing fruit quality and ripeness (Zhang, Chen, Wu, Kou, & Xue, 2024). Total soluble solids (TSS) ^o^Brix increased in all samples during 20–40 days and subsequently decreased until the end of storage period. The Total soluble solids (TSS) of 11.5 at 0 day increased to 12.6 at 20th day in case of P1TI (wooden box + control), 12.4 in P1T3 (wooden box + KMnO_4_), 12.7 in P2T1 (CFB + control), 12.3 in P2T3 (CFB + KMnO_4_), 12.3 in P3T1 (shrink wrap + control) and 12.2 in P3T3 (shrink wrap + KMnO_4_). In contrast, Total soluble solids (TSS) of P1T2 (wooden box +1-MCP), P2T2 (CFB +1-MCP) and P3T2 (shrink warp +1-MCP) increased slightly to 12.7, 12.6 and 12.4 at 40th day of storage and subsequently decreased to 9.4, 9.9 and 10.2 respectively at the end of the storage period. These results are in accordance with Abbès et al., 2011 who reported that higher Total soluble solids (TSS) increment during storage might be due to the hydrolysis of starch into sugars by the action of enzymes. However, decreased Total soluble solids (TSS) with respect to storage may be due to faster utilization of carbohydrates in respiration, senescence and oxidation processes (Ahmad, Zaidi and Arshad, 2021a, Ahmad, Zaidi and Arshad, 2021b). Total soluble solids (TSS) content in apples (Gala Redlum, Red Velox, Super Chief) increased up to 30 days in ambient storage and 60 days in cold storage, then declined thereafter. These findings are consistent with previous records of Wani et al. (2024).

Acidity presumably indicates the tartness of the fruit & contributes to a more pronounced tangy flavor and balance sweetness, affecting the overall sensory experience of the fruit (Kim, Chun, Suh, & Sung, 2023).The titrable acidity (mallic acid %) value of all treated and untreated apples showed little decrease throughout the storage period of 160 days. The acidity (mallic acid %) value of 0.38 at 0 day decreased to 0.18 at 160th day in case of P1TI (wooden box + control), 0.23 in P1T3 (wooden box + KMnO_4_), 0.25 in P2T1 (CFB + control), 0.26 in P2T3 (CFB + KMnO_4_), 0.27 in P3T1 (shrink wrap + control) and 0.28 in P3T3 (shrink wrap + KMnO_4_). However, P1T2 (wooden box +1-MCP), P2T2 (CFB +1-MCP) and P3T2 (shrink warp +1-MCP) showed the acidity (mallic acid %) value of 0.27, 0.28 and 0.30 at 160^h^ day of storage respectively. Reports showed that decline in titrable acidity (mallic acid %) values is caused due to the utilization of acid as a partial substrate for respiration for this reason, the decrease in acidity was generally more pronounced later in the shelf life (Jahangir et al., 2022). This study is consistent with findings of Ahmad, Zaidi and Arshad, 2021a, Ahmad, Zaidi and Arshad, 2021b who observed that the acidity of apples decreased from 0.163 ± 0.003 % malic acid to 0.081 ± 0.001 % during storage at ambient temperature. In line with the findings of Li et al. (2023), respiration devoured the organic acids, resulting in a decrease in the titratable acidity levels in the Ralls, Delicious Qinguan, Fuji and Cattle apple cultivars. 1-MCP applied, compete-out ethylene in binding to the receptor sites, thus inhibiting the formation of ethylene receptor complexes. In this way, the ethylene-induced fruit ripening process is blocked hence, 1-MCP application delays the onset of the rise in ethylene production (Gomez, Sebastian, Anjali, Joseph, & Maneesha, 2023) whereas, shrink wrap packaging further modifies the atmospheric conditions inside the cover by providing protection against moisture and oxygen uptake which results in reduced metabolic activities (Maqbool et al., 2024).

#### 3.2.1 Effect of 1-MCP, KMnO_4_ and packaging material on textural attributes

**Fig. 4:**
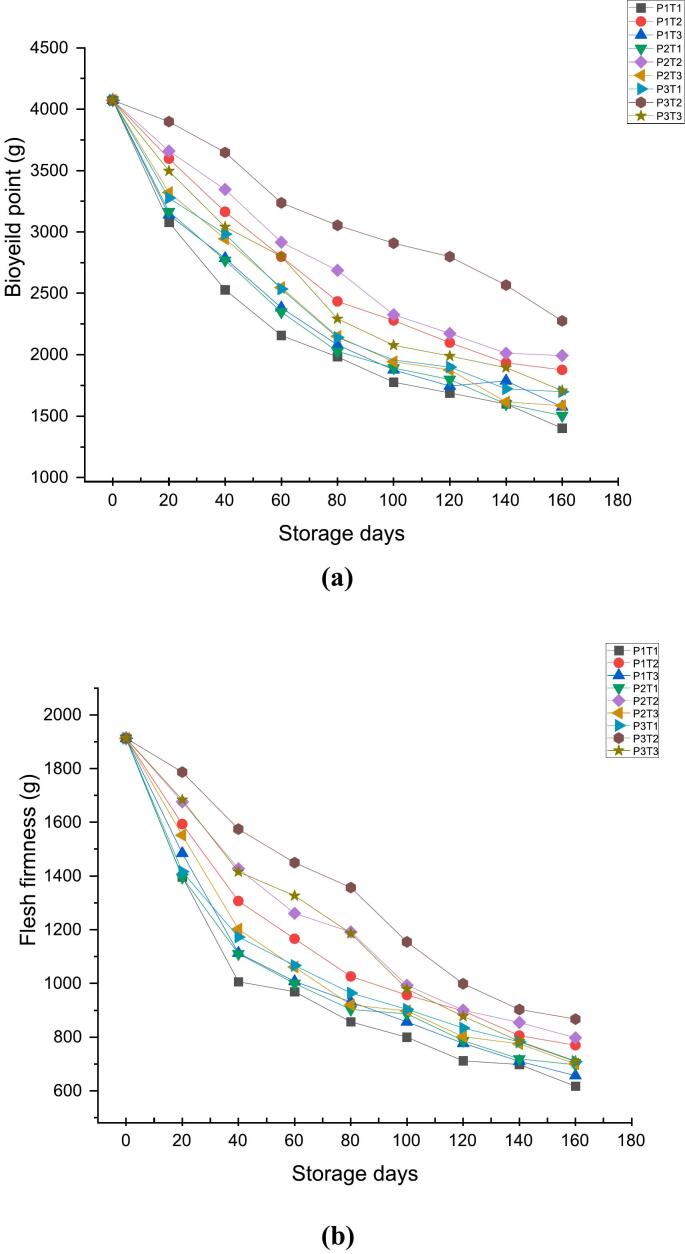

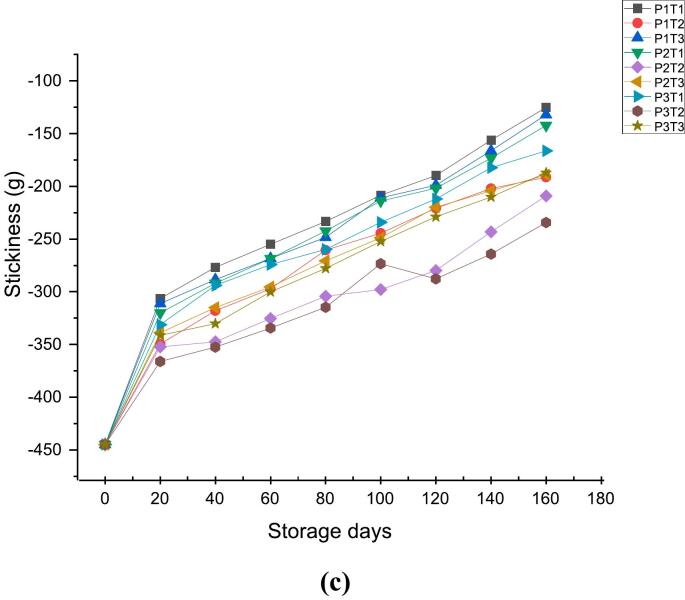
4a: Effect of 1-MCP, KMnO_4_ and packaging material on bio yield point (g). 4b: Effect of 1-MCP, KMnO4 and packaging material on Flesh firmness (g). 4c: Effect of 1-MCP, KMnO4 and packaging material on Stickiness (g).

Bio yield point is a critical parameter for assessing the fruit's mechanical integrity during storage and transport (Kumar, Janghu, & Chaudhary, 2024). Fig. 4. 4aa, 4. 4ab & 4. 4ac, shows the effect of ethylene scrubbers and packaging on Textural attributes of apples during 160 days of storage. The bio yield point of all the treated and control samples decreased gradually with the increase in storage duration, which may be due to the degradation of pectin by enzyme endopolygalacturanase (Liu, Renard, Rolland-Sabaté, Bureau, & le Bourvellec, 2021). However, the rate of reduction of bio yield point from an initial value of 4078.8 at 0 day was found to be 1401.3 at 160th day in case of P1TI (wooden box + control), 1574.3 in P1T3 (wooden box + KMnO_4_), 1504.2 in P2T1 (CFB + control), 1585.2 in P2T3 (CFB + KMnO_4_), 1698.2 in P3T1 (shrink wrap + control) and 1708.2 in P3T3 (shrink wrap + KMnO_4_). However, in P1T2 (wooden box +1-MCP), P2T2 (CFB +1-MCP) and P3T2 (shrink warp +1-MCP) bio yield point of 1876.2, 1993.4 and 2275.7 were observed at 160th day of storage respectively. Prior records of Shazia (2023) are in agreement with these findings who reported a decreasing trend in the bio-yield point of Red chief apples during 30 days of ambient storage. The study by Aaruba (2023) also found that the bio yield point of Red Delicious apples decreased with an increase in storage duration of 160 days under ambient conditions.

Similarly, the flesh firmness of all samples decreased throughout the storage period at ambient condition, however, from an initial value of 1913.1 at 0 day, the flesh firmness decreased to 617.4 in P1TI (wooden box + control), 656.5 in P1T3 (wooden box + KMnO_4_), 697.5 in P2T1 (CFB + control), 700.3 in P2T3 (CFB + KMnO_4_), 709.2 in P3T1 (shrink wrap + control) and 711.2 in P3T3 (shrink wrap + KMnO_4_). In contrast, the P1T2 (wooden box +1-MCP), P2T2 (CFB +1-MCP) and P3T2 (shrink warp +1-MCP) showed flesh firmness of 769.9, 797.6 and 867.3 respectively at the end of the storage period. According to Zhao et al. (2020), the loss of the primary cell wall and middle lamella structures, as well as the pectin degradation caused by the high activity of the enzyme endopolygalacturanase, are associated with the deterioration of firmness. This contradicts the results of previous study (Li et al., 2019) that found that from 105 days after full bloom (DAFB), both firmness and pectin content rapidly declined, with the reduction being more pronounced in the ‘Fuji’ variety than in the ‘Qinguan’ variety. Contrary to the literature (Sharma & Saini, 2021) all samples began to lose firmness by the 6th day of storage, likely due to hydrolytic enzymes like β-D-glucosidase degrading pectic substances in guavas. The study (Liu et al., 2023a, Liu et al., 2023b) observed that the apple cultivars ‘Jiguan,’ ‘Yindu,’ and ‘Qinguan’ experienced rapid texture loss, evidenced by a faster decline in flesh firmness compared to other varieties.

Further, from an initial value of −444.9 at 0 day the stickiness of the fruit showed sharp decline of −125.2 in P1TI (wooden box + control),-132.1 in P1T3 (wooden box + KMnO_4_),-142.2 in P2T1 (CFB + control), −189.2 in P2T3 (CFB + KMnO_4_), −166.3 in P3T1 (shrink wrap + control) and – 187.2 in P3T3 (shrink wrap + KMnO_4_). In contrast, the P1T2 (wooden box +1-MCP), P2T2 (CFB +1-MCP) and P3T2 (shrink warp +1-MCP) showed the stickiness of −191.3, −209.1 and – 234.4 at 160th day of storage respectively. Results of this study are in accordance with the previous studies of Liu et al., 2023a, Liu et al., 2023b who reported that the degree of methyl esterification is altered during ripening causing dissociation of pectin chains and reducing cohesion between the cells in the cell wall hence, leading the stickiness to get decreased. Pectin solubilization in the intermediate lamellae between adjacent cells is the cause of the stickiness reduction that has been seen during storage reported by Frempong et al., 2022. Previous studies of Cybulska et al., 2015 found that stickiness decreases during ripening as pectin breakdown weakens cell cohesion, leading to increased cell separation and reduced stickiness.

1-MCP along with shrink wrap packaging maintains the textural characteristics of apples as 1-MCP is known to curtail the decomposition of the cell wall and suppress the cell wall degradation-linked genes and their associated enzyme activities (Win, Yoo, Naing, Kwon, & Kang, 2021) whereas, shrink-wrap enhances the efficiency of 1-MCP by creating a barrier between the fruit and the outside atmosphere and prevents 1-MCP (gas) to escape Jama (2023).

### 3.3 Effect of 1-MCP, KMnO_4_ and packaging material on organoleptic properties

**Fig. 5:**
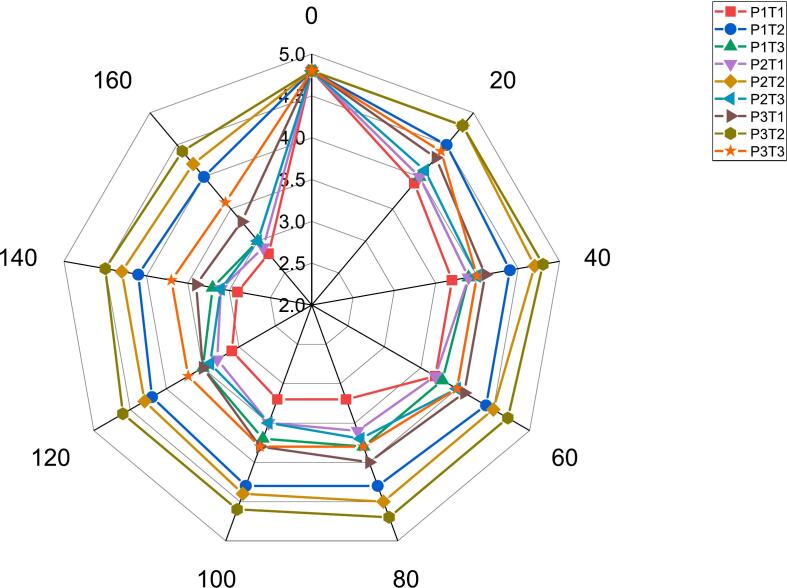
Effect of 1-MCP, KMnO_4_ and packaging material on organoleptic properties.

Sensory evaluation helps to identify preferred characteristics and detect any quality changes over time, thereby influencing both the perceived value of the apples and their suitability for extended storage. Fig. 5a, indicates the effect of 1-MCP, KMnO_4_ and different packaging materials on organoleptic properties. Both treated and control samples record sharp decline in sensory evaluation during 160 days of storage. At 0 day, the sensory score was analysed as 4.8 which declines to 2.8 in P1TI (wooden box + control), 3.0 in P1T3 (wooden box + KMnO_4_), 2.9 in P2T1 (CFB + control), 3.0 in P2T3 (CFB + KMnO_4_), 3.3 in P3T1 (shrink wrap + control) and 3.6 in P3T3 (shrink wrap + KMnO_4_) respectively. However, P1T2 (wooden box +1-MCP), P2T2 (CFB +1-MCP) and P3T2 (shrink warp +1-MCP) showed the sensory score of 4.0, 4.2 and 4.4 at 160th day of storage respectively. Ahmad, Zaidi and Arshad, 2021a, Ahmad, Zaidi and Arshad, 2021b also found that prolonged storage led to decreased panellist preference for apples due to changes in taste, texture, and aroma, highlighting the importance of monitoring sensory attributes to maintain quality and shelf life. Contrary with the study of Wani et al. (2024), the sensory score decreased to “like moderately” after two months of ambient storage and after four months and ten days of cold storage. Thus, shrinkwrap+1-MCP (P_3_T_2_) showed superiority in maintaining the overall acceptability than other treatment combinations throughout the storage period. These observations are in conformity to the findings of Tomala et al. (2022).

## 4 Conclusion

The results of the present work enabled us to conclude that post-harvest application of 1-MCP along with shrink wrapped packaging showed superiority in enhancing the shelf-life of apple cv. Golden delicious followed by shrink wrap+KMnO_4_. From the outcome of the study, it is revealed that application of 1-MCP along with shrink wrapped packaging in Golden delicious apples better retained the fruit firmness lb./sq. (10.7), TSS ^**0**^Brix (10.2), juice recovery % (37.12), while reduced the rot % (5.1), physiological loss in weight %(10.34) etc. Hence, it was possible to summarize that treated fruits remained in fair to good quality upto 160 days of ambient storage thereby maintained the quality attributes of the fruit which reduces the post-harvest losses and enhance the shelf life of the fruit. These findings offer a practical solution for reducing post-harvest losses and extending the shelf life of apples in ambient conditions.

## CRediT authorship contribution statement

**Aaruba Maqbool:** Writing – review & editing, Writing – original draft. **Mushtaq Ahmad Beigh:** Funding acquisition, Formal analysis, Conceptualization. **Syed Zameer Hussain:** Supervision. **Tashooq Ahmad Bhat:** Writing – review & editing, Writing – original draft. **Imtiyaz Ahmad Zargar:** Supervision. **Shazia Akhter:** Formal analysis. **Nazrana Wani:** Software. **Tahiya Qadri:** Resources, Software.

## Declaration of competing interest

The authors declare that they have no known competing financial interests or personal relationships that could have appeared to influence the work reported in this paper.

## Data Availability

Data will be made available on request.

## Appendix A Supplementary data

Supplementary material Image 1
