# Supplementary figures and images for: Effect of 1-MCP and KMnO4 treatments with different packaging on quality preservation of golden delicious apples
Source: Food Chem X. 2024 Aug 24;23:101768. doi: 10.1016/j.fochx.2024.101768 (PMC11402158; doi:10.1016/j.fochx.2024.101768)

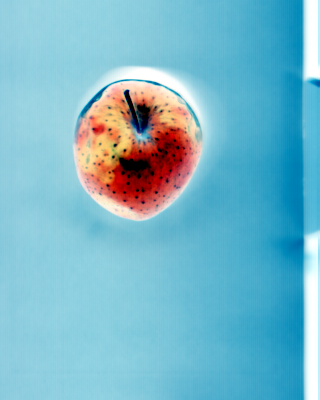

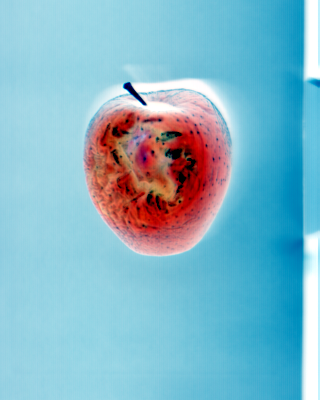

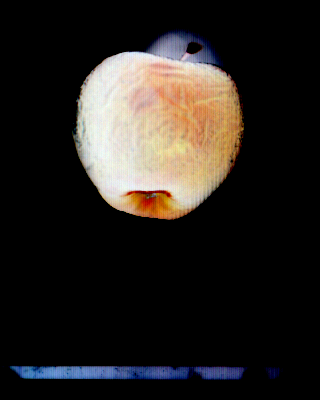

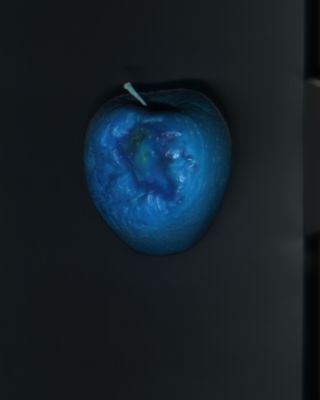

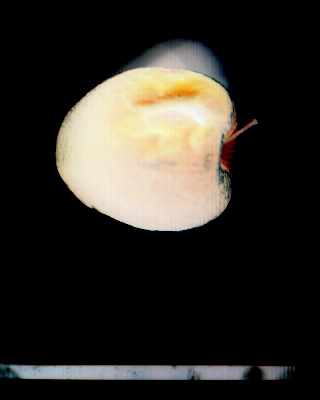

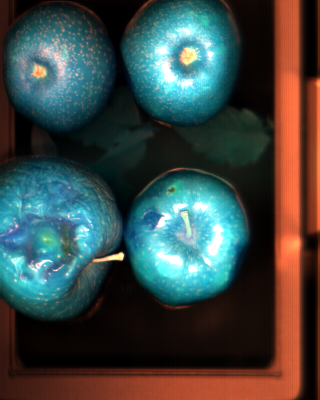


Fig 1S: Hyper-spectral imaging showing early surface damage of apples during storage

Supplement: Supplementary file 1 — Supplementary material [file mmc1.docx]
